# Acute Glomerulonephritis as a Paraneoplastic Syndrome Secondary to Urachal Adenocarcinoma: An Unknown Entity

**DOI:** 10.7759/cureus.60106

**Published:** 2024-05-11

**Authors:** Carmen G Bermúdez Barrientos, Marisol Ramos Portales, Edna T Mendoza Villalobos, Brizio Moreno Jaime

**Affiliations:** 1 Medical Oncology, Institute for Social Security and Services for State Workers Regional Hospital, Leon, MEX; 2 General Surgery, Institute for Social Security and Services for State Workers Regional Hospital, Leon, MEX; 3 Nephrology, Institute for Social Security and Services for State Workers Regional Hospital "Dr. Valentín Gómez Farías", Guadalajara, MEX

**Keywords:** papillary variant, malignant glandular epithelial neoplasm, paraneoplastic syndrome, glomerulonefritis, urachal adenocarcinoma

## Abstract

Urachal carcinoma is an uncommon malignancy with a peculiar biomolecular characterization and therefore a complex approach. It was incorporated by the World Health Organization in 2004 in the tumors of the urinary system classification. This neoplasm is generally diagnosed in advanced stages. The standard treatment is surgical, however, due to the rarity and relatively late clinical manifestation of urachal carcinomas, the survival data are mostly case reports, as well as information about medical-surgical treatment based on evidence.

The data used were extracted from both the physical and electronic clinical records. Among atypical presentations reported in the literature, we report a case of urachal adenocarcinoma with simultaneous glomerulonephritis as a paraneoplastic syndrome of which there is no report to date. Surgery was carried out in our patient, unfortunately with lifetime morbidity from kidney function replacement secondary to kidney function damage by glomerulonephritis, despite previous immunosuppression treatment for rapidly progressive glomerulonephritis. It is worth mentioning that if the initial diagnosis represents a clinical challenge, treatment is even more complex, given the little information that currently exists about it.

Urachal carcinoma is a diagnostic and treatment challenge. Up to now, surgery has been the treatment of choice in localized or locally advanced disease, however, with a high morbidity for the patient.

## Introduction

Urachal carcinoma is an infrequent neoplasm that originates from the urachal ligament, with an approximate prevalence between 0.01% to 0.02% of all cancers in adults worldwide, more frequent between the 5th and 6th decades, with a male-to-female ratio of 1.4-1.6:1 [1-2]. It was first described by Hue and Jacquin in 1863 [3] and incorporated into the World Health Organization in the classification of urinary tract tumors in 2004. The urachus is an embryonic vestige that joins the allantois and the fetal bladder, where urine drains during the first trimester of pregnancy. After its obliteration, it consists of a fibromuscular canal, known as the median umbilical ligament. It is located in a preperitoneal pyramidal space and is formed by striated structures that connect the anterior arched roof of the bladder with the umbilicus (belly button). Its configuration consists of three layers: smooth muscle, connective tissue in the middle, and at last urothelium. It is worth mentioning that neoplasms can arise in any of these portions [4]. The most recurrent histological type reported is adenocarcinoma [1]. This unusual hidden location is the reason why symptoms such as hematuria and pain come into sight in advanced stages, resulting in about a third of patients having metastases at the time of diagnosis, which is associated, as expected, with a poor prognosis even in the short term [5]. Patients with locally advanced or metastatic disease have a median survival of 12 to 24 months and a 5-year survival rate of 43% [6]. Urachal adenocarcinomas appear to be a distinct entity at the molecular level with a closer resemblance to colorectal adenocarcinoma than to urothelial carcinomas [7]. The standard treatment is essentially surgical, however, due to the rarity and relatively late clinical manifestation of urachal carcinomas, survival data are scarce as is information about evidence-based medical and surgical treatment [2].

## Case presentation

The case of a 41-year-old male patient is presented, who had the following oncological family history: mother with ovarian cancer. He denied any personal pathological history. His current condition began in December 2019 with intermittent macroscopic hematuria. As part of the diagnostic approach, a computed tomography-guided renal biopsy was performed in March 2021, reporting the following in the histopathology report: focal and segmental glomerulonephritis, chronic glomerular hypoperfusion, active and chronic tubulointerstitial nephritis with acute focal tubular lesion and with moderate epithelial regenerative changes; interstitial fibrosis grade 2 (30-40%), moderate atherosclerosis. The IgG, IgA, C1q, C3c, C4c, Kappa, and Lambda immunophenotype tests were negative, however, IgM was positive in the form of mesangial filaments, and albumin with a linear pattern.

Treatment was given with boluses of methylprednisolone. Due to impaired renal function, peritoneal dialysis was started and macroscopic hematuria continued intermittently. The patient presented hyporexia and weight loss of 5 kg, without presenting uremic syndrome. The specialty of Nephrology concluded glomerulonephritis** **secondary to paraneoplastic syndrome. A computerized axial tomography urography was carried out in June 2021, observing the following: rounded intravesical lesion suggestive of a vesicourachal diverticulum. Cystoscopy was performed in July 2021. It found a 3.5 cm solid tumor in the bladder fundus. A biopsy was taken, whose histopathological report resulted in the following: malignant glandular epithelial neoplasm, moderately differentiated adenocarcinoma with villous and sieve areas, predominantly intestinal areas. PSA and CD31 were performed, which were negative. Endoscopy and colonoscopy were normal.

**Figure 1:**
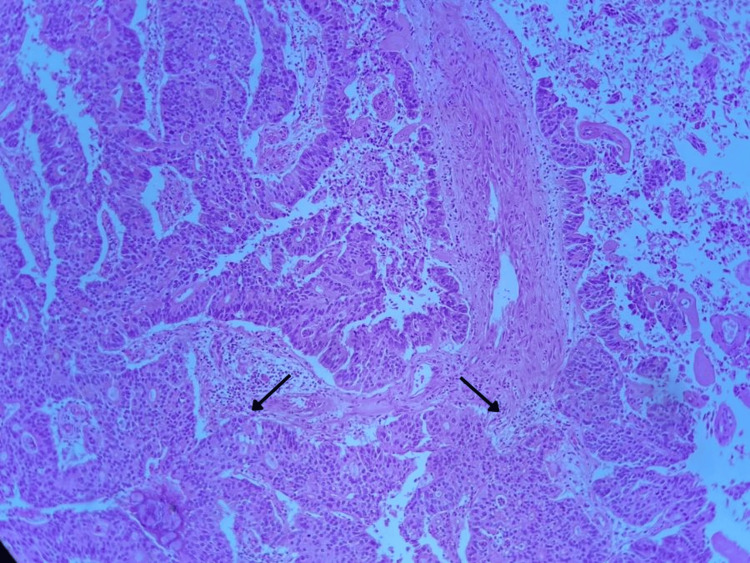
Histological slide. Glandular formations that infiltrate the basement membrane into the surrounding connective tissue and smooth muscle. Non-urotelial involvement.

**Figure 2:**
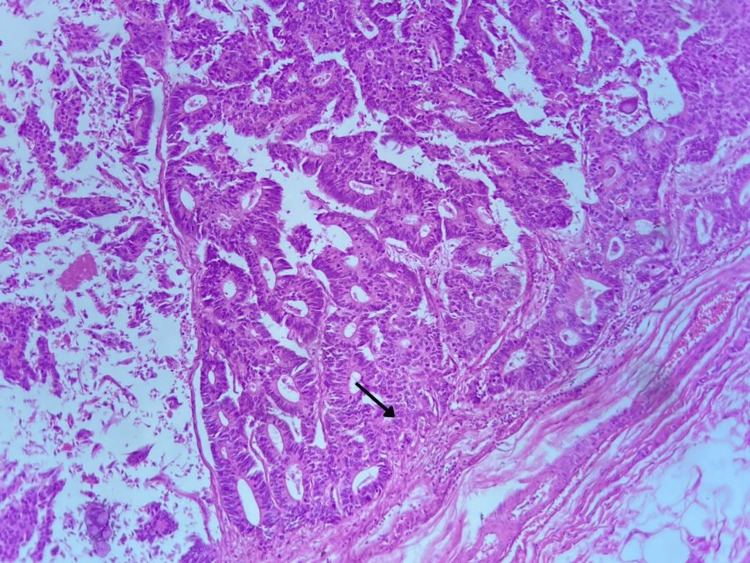
Histological slide. Atypical glandular cellularity with loss of nuclear polarity in relation to the glandular basement membrane, and, in addition, giving the appearance of an enteric morphology.

**Figure 3:**
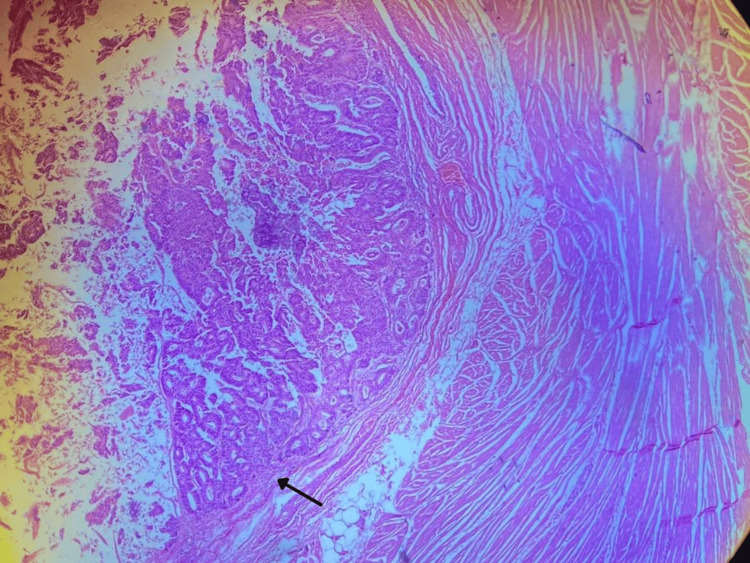
Histological slide. Glandular formations with atypical cellularity that spare the basement membrane and attached structures.

**Figure 4:**
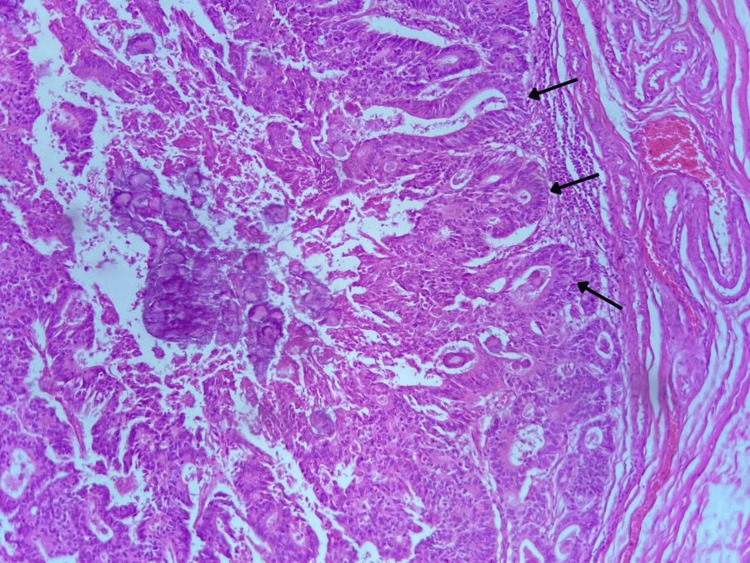
Histological slide. Glandular architecture lined by pseudostratified malignant epithelium (intestinal type).

The patient was scheduled in October 2021 for tumor resection, however, non-specific pneumonia occurred, so the procedure was deferred. Surgery was rescheduled for February 2022, performing bilateral nephrectomy, radical bladder and prostate resection. He was switched to hemodialysis due to a cavity not suitable for peritoneal dialysis after the surgical procedure. The definitive histopathological report evidenced the following: grade 2 moderately differentiated adenocarcinoma, papillary variant, of the urachus, size 4 cm, without lymphovascular infiltrate (Figures 1-4). 

The patient currently remains under the surveillance of the Urology and Medical Oncology services and has completed 1 year of progression-free survival.

## Discussion

Urachal neoplasms constitute a rare entity both epidemiologically speaking and in terms of their nature and behavior, without leaving behind the potential aggressiveness. Their behavior is not as typical adenocarcinoma or urothelial carcinoma [8].

While in extrauterine life the urachus usually constitutes an obliterated fibrous structure, which is found in the retzius space and that functionally no longer performs any important role, during pregnancy, specifically prior to its obliteration, approximately between the 4th and 5th month of gestation, it functions as an allantoic stem through which fetal urine drains [9].

No reliable risk factors have been identified for this entity. At some point, it was considered that deficient obliteration of the urachus predisposed to the development of urachal adenocarcinoma. However, there are no clinical trials that have demonstrated this relation. It is also useful to consider that up to 30% of the human urachus may not be completely occluded before birth and persist in adulthood, contrasting that fact with the low incidence of urachus carcinoma.

The characterization of urachal adenocarcinomas is intriguing from a molecular point of view since they share a significant affinity with colon adenocarcinoma.

Primary urachal tumors harbor recurrent KRAS, NRAS, BRAF, APC, TP53, NF1, and/or SMAD4 mutations but generally lack TERT promoter and PIK3CA mutations [10].

In the same way as the peculiarity of the characterization of urachal adenocarcinomas, the clinical presentation constitutes a diagnostic challenge at the beginning, due to both the anatomical location of this structure, as well as the wide variability in the manifestation of symptoms (hematuria, dysuria, mucosuria, abdominal pain, among others like pollakiuria, urinary tract infection, umbilical discharge, and nausea) and the temporality of the same. As an effect of this, the establishment of a definitive diagnosis and therefore the start of treatment is delayed in most cases, representing a worse prognosis [11].

The first diagnostic criteria were proposed by Wheeler and Hill in the 1950s [12]. Two staging systems are currently commonly used for urachal cancer: one was proposed by Sheldon et al. in 1984 [13], and the other simplified system was proposed by the Mayo Clinic in 2003, both are the main independent predictors of prognosis. However, according to Yu et al., serum, immunohistochemical, and genetic markers showed little potential for risk classification [5].

Among the histology subtypes, the most frequently reported is the mucinous (43-75%), followed by intestinal (24%) and mixed types (10%).

Among the atypical presentations reported in the literature, we report the case of urachal adenocarcinoma with simultaneous glomerulonephritis as a paraneoplastic syndrome of which there have been no reports to date. 90% of urachal carcinomas are adenocarcinomas, as is the case we report here, clinically manifested at the beginning with hematuria in 73% of cases. No glomerulonephritis associated with this entity has been described before, however, it has a prevalence as a paraneoplastic syndrome of up to 10%, especially in lung cancer, followed by gastric and renal cancers. 

Regarding the diagnostic approach, cystoscopy locates the tumor in most cases (89-94.1%).

In our case, despite the rare presentation of glomerulonephritis as a paraneoplastic syndrome in this entity, the diagnosis and surgical treatment were done and the patient has completed 1 year of progression-free survival. The choice of adjuvant or neoadjuvant treatment, either chemotherapy and/or radiotherapy or even targeted therapy and immunotherapy is based on isolated case reports. Nevertheless, the treatment of choice is essentially surgical, which was carried out in our patient with encouraging results so far [14]. The ideal treatment in solid tumors is tumor ablation [9] and immunosuppressive treatment for rapidly progressive glomerulonephritis. However, the extended partial cystectomy or radical cystectomy with en bloc resection of the urachal tumor, the entire urachus, umbilicus, and bilateral pelvic lymph node dissection is the recommended surgical approach [4,6]. Nevertheless, the role of bilateral pelvic lymphadenectomy is still controversial in not improving overall survival and is associated with a high complication rate and only 17% of lymph node positivity [11]. Surgery was performed in our patient, unfortunately with lifetime morbidity of renal function substitution, due to bilateral nephrectomy and previous renal function damage secondary to glomerulonephritis. Surgery is the gold standard for localized disease [14]. 

It is recommended that, although there is no defined protocol, patients with the presence of a positive margin, lymph node metastasis, peritoneal involvement, and without navel resection, receive 4-6 cycles of chemotherapy. Some regimens based on 5-Fu have shown up to 30-40% radiographic response rates [6]. 

It is worth mentioning that if the initial diagnosis represents a clinical challenge, the treatment is even more complex given the current lack of information from prospective randomized trials in this regard [10], not to mention its aggressiveness, considering that the estimated 5-year survival rate does not reach 50% [14].

## Conclusions

Urachal carcinoma is a diagnostic and treatment challenge. So far surgery has been the treatment of choice in localized or locally advanced disease, with a 5-year survival rate of 43%, but with a high morbidity rate for the patient. 

It is important to integrate the information that exists up to now in order to obtain the best results in patients with this diagnosis. However, due to the low incidence, the significance of reporting data that is generated in this regard is substantial.
